# Cohort profile: the Hlabisa pregnancy cohort, KwaZulu-Natal, South Africa

**DOI:** 10.1136/bmjopen-2016-012088

**Published:** 2016-10-17

**Authors:** Terusha Chetty, Claire Thorne, Frank Tanser, Till Bärnighausen, Anna Coutsoudis

**Affiliations:** 1 Wellcome Trust Africa Health Research Institute, KwaZulu-Natal, South Africa; 2 Department of Public Health Medicine, School of Nursing and Public Health, University of KwaZulu-Natal, KwaZulu-Natal, South Africa; 3 UCL Institute of Child Health, University College London, London, UK; 4 Department of Global Health and Population, Harvard School of Public Health, Boston, Massachusetts, USA; 5 Institute of Public Health, Medical School, University of Heidelberg, Heidelberg, Germany; 6 Department of Paediatrics and Child Health, Nelson R. Mandela School of Medicine, University of KwaZulu-Natal, KwaZulu-Natal, South Africa

**Keywords:** pregnancy cohort, antiretroviral therapy

## Abstract

**Purpose:**

The Hlabisa pregnancy cohort was established to evaluate the effectiveness of prevention of mother-to-child transmission (PMTCT) guideline revisions. The objectives of the Hlabisa pregnancy cohort are to: (1) provide cohort-level information on maternal health up to 6 weeks postpartum in a high HIV prevalence setting; and to (2) evaluate aspects of PMTCT care that have policy relevance.

**Participants:**

The pregnancy cohort is located in primary health clinics in the Hlabisa subdistrict of rural KwaZulu-Natal, South Africa. Baseline data collection between 2010 and 2014 has been completed with the enrolment of 25 608 pregnancies; age ranged from 15–49 years. Pregnant women were assessed during routine antenatal visits: first visit, follow-up 1 week later, 32 weeks (HIV test), infant delivery and 6 weeks postpartum. Demographic, pregnancy, clinical, laboratory and HIV data were collected through Department of Health interviews, laboratory tests and routine data linkage. Treatment data for HIV-infected pregnant women were linked to the Africa Centre Hlabisa HIV Treatment and Care Programme for detailed antiretroviral therapy (ART) history and laboratory tests.

**Findings to date:**

The proportion of women initiated on ART post-2013 were higher (n=437; 100%) than pre-2013 (n=768; 84.2%). The proportion of women in care at 6 weeks (73.8%) was also higher post-2013 relative to earlier years (58.5%). The majority of HIV-infected pregnant women were either on lifelong ART or ART prophylaxis; pre-2013, ∼ 9.6% of women were not on any ART. Pregnancy viral load monitoring was inadequate.

**Future plans:**

This cohort will be used to: (1) determine HIV acquisition risk during pregnancy and postpartum; (2) determine the effect of HIV and ART on birth outcomes; (3) examine the effect of pregnancy on virological response to ART; and (4) characterise the effect of sequential pregnancies on access to clinical care, response to prolonged ART and birth outcomes.

**Strengths and limitations of this study**

- The key characteristic of the Hlabisa pregnancy longitudinal cohort is size and ability to model the impact of the HIV programme on the community through linkage of Africa Centre's population level data with clinical, pregnancy and HIV data.
- Follow-up of HIV-infected mothers is crucial to monitor adherence to antiretroviral therapy and disease progression.
- Assessment of long-term maternal outcomes may be limited by high population mobility and use of routine health sector data.

## Introduction

Prevention of mother-to-child transmission (PMTCT) of HIV using antiretroviral drugs can nearly eliminate vertical HIV transmission and increase maternal survival.^1^ However, poor delivery of any of the steps in PMTCT results in cumulative losses of pregnant women, raising infant HIV transmission risk.^2^ The PMTCT ‘cascade’ highlights the optimum PMTCT sequence: HIV counselling and testing at the first antenatal visit; CD4 measurement; antiretroviral therapy (ART) initiation and adherence; and early infant HIV testing,^2^ or after cessation of breast feeding and infant HIV test at 18 months.^3^

The Hlabisa HIV Treatment and Care Programme (HHTCP), described previously,^4^ was a partnership between the South African Department of Health (DoH) and the Wellcome Trust funded Africa Centre for Population Health (Africa Centre). In January 2010, the Africa Centre established a pregnancy cohort aligned with the HHTCP objectives to monitor PMTCT for feedback to funders. A further objective is to use this cohort to determine clinical markers related to pregnancy in this high HIV prevalence setting. The pregnancy cohort can be linked with the Africa Centre Demographic Information System (ACDIS), which has demographic and health data through population-based longitudinal surveillance of ∼90 000 people in 12 000 households, including accurate longitudinal HIV incidence, HIV prevalence and ART coverage estimates for this subpopulation since 2003.^5^ ^6^ Hence, demographic and health factors related to the PMTCT programme success can be monitored.

### Cohort description

#### Setting

**Figure 1:**
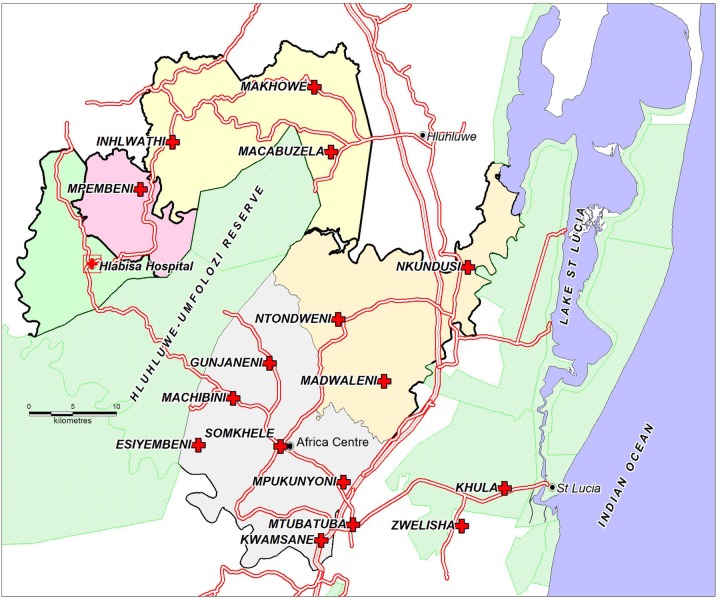
Africa Centre surveillance area showing the position of Hlabisa Hospital with an on-site clinic and 16 peripheral clinics in the Hlabisa subdistrict, KwaZulu-Natal, South Africa. The Hlabisa subdistrict encompasses the area to the bottom-right of the map which includes Mtubatuba and Zwenelisha clinics.

The predominantly rural Hlabisa subdistrict of uMkhanyakude in northern KwaZulu-Natal, South Africa has a population of ∼228 000.^5^ There are 17 nurse-led primary healthcare clinics (PHC) with a primary level district hospital (Hlabisa Hospital), which handles most of the deliveries. Six DoH clinics and 40% of patients are located within ACDIS (figure 1). Household surveillance began in January 2000. Routine household visits were conducted biannually, and since 2012 three times a year, to collect information about births, deaths and migrations. The number of resident and non-resident females aged 15–49 years in ACDIS from 2010 through 2014, were respectively: 65 454 (27.3%); 65 352 (27.5%); 65 889 (27.7%); 65 092 (27.8%); and 62 705 (27.9%).

##### Ethics

Owing to the dynamic movement of patients within antenatal clinics with varying entry times, including delivery, reliable written informed consent for pregnancy data linkage with ACDIS was challenging. Additionally, ACDIS consent is at household level, with either the household head providing verbal consent for surveys to be conducted, or a proxy in the absence of the household head. We therefore requested a waiver of written informed consent for pregnancy and ACDIS data linkage from the University of KwaZulu-Natal Biomedical Research Ethics Committee (E134/06). Instead, women attending antenatal clinics gave verbal consent to link their details with ACDIS data.

### Who is in the cohort?

From 1 January 2010 to December 2014, all pregnant women attending antenatal care (ANC) for the first time at PHC clinics in the subdistrict entered the cohort automatically. Data were initially collected from all 17 PHC clinics in Hlabisa up to 2012; thereafter, since funding was restricted, the data collection focal point became six PHC clinics within ACDIS and one clinic located just outside the surveillance due to the proximity to the national road.

**Figure 2:**
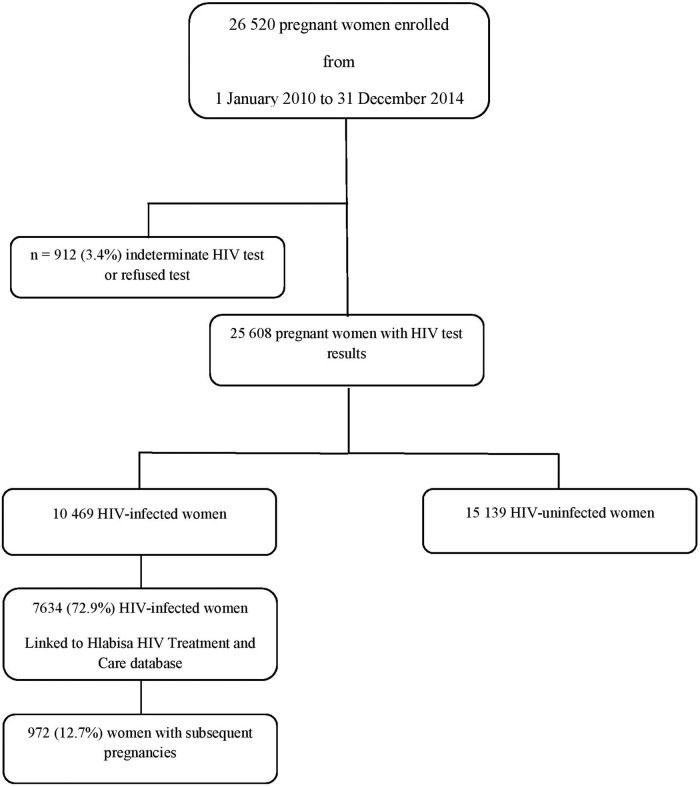
Flow diagram of the Hlabisa pregnancy cohort.

The cohort has completed enrolment for 25 608 pregnancies recorded from 1 January 2010 until 31 December 2014 (figure 2). A secure database system was developed to capture data on women from the first antenatal visit through delivery up to 18-month infant follow-up.

Data from 7634 HIV-infected pregnant women have been linked with the Africa Centre HHTCP database to provide details on HIV treatment and monitoring, including postpartum follow-up.^4^ All pregnant women were offered HIV counselling and testing at their first visit. Women who disclosed that they were HIV-infected (ie, known HIV status before pregnancy) were asked about prior PMTCT or ART and if they had started lifelong ART.

Pregnancies of HIV-infected women were categorised according to ART initiation timing: (1) Already on lifelong ART prior to first antenatal visit; (2) Started lifelong ART within 6 months of the first antenatal visit; and (3) Not on lifelong ART during pregnancy (as assessed at delivery). For those patients already on lifelong ART prior to the first antenatal visit, we report the ART regimen prescribed within 6 months of the first visit.

#### Clinical care and follow-up

Until 2013, pregnant women first testing HIV negative had repeat HIV testing recommended at or after 32 weeks gestation; the 2013 HIV guidelines recommend repeat HIV testing for HIV-uninfected pregnant women three monthly throughout breast feeding.^7^ The 2010 and 2013 PMTCT guidelines are summarised in table 1. Since 2015, South Africa has followed the WHO recommendation to initiate all pregnant HIV-infected women on lifelong ART, regardless of CD4 count (Option B+).^8^ ^9^ Before 2010, the first-line regimen recommended for patients with a CD4 count ≤ 200 cells/mm^3^ or WHO stage 4 was stavudine (d4T), lamivudine (3TC) and either efavirenz (EFV) or nevirapine (NVP).^10^ The 2010 ART adult guidelines recommended that patients already on a d4T-based regimen continue on this treatment if well tolerated, with early switch to tenofovir (TDF) with any toxicity.^11^ Tenofovir was rolled out according to a phased implementation plan. Pregnant patients were prioritised if they were newly initiating ART in April 2010. By April 2011, d4T to TDF switches were prioritised; hence, there was a lag with d4T regimen changes for patients who were initiated prior to April 2010 if side effects were not severe (Naidu KK (HHTCP Lead, 2016)).

**Table 1:** South African prevention of mother-to-child transmission guidelines

Regimen	2010 guidelines	2013 guidelines
PMTCT prophylaxis (CD4 > 350 and WHO stage 1/2)	Antenatal zidovudine (AZT) from 14 weeks; Intrapartum single-dose nevirapine (sdNVP), 3-hourly AZT; Postpartum single dose of TDF+emtricitabine (FTC)	TDF, 3TC/FTC, EFV to be initiated as soon as pregnancy is diagnosed (if no active psychiatric illness or history of renal disease) to be continued through the postnatal period until 1 week after complete cessation of breast feeding (WHO Option B)^8^
	*Infant regimen*: NVP at birth and then daily for 6 weeks, continued as long as any breast feeding; if formula-fed, infant NVP should stop at 6 weeks	*Infant regimen*: NVP at birth and then daily for 6 weeks; if the mother on AZT regimen, the infant should receive NVP at birth, then daily for 6 weeks to be continued until 1 week after complete cessation of breast feeding
Lifelong ART (CD4 ≤350 or WHO stage 3/4)	TDF, 3TC/FTC, EFV	TDF, 3TC/FTC, EFV
	*Infant regimen*: NVP at birth and then daily for 6 weeks irrespective of feeding choice	*Infant regimen*: NVP at birth and then daily for 6 weeks

ART, antiretroviral therapy; AZT, zidovudine; EFV, efavirenz; FTC, emtricitabine; 3TC, lamivudine; NVP, nevirapine; TDF, tenofovir.

The approximate proportion of patients on d4T regimens in the HHTCP were as follows: (1) by December 2012, ∼20–30% of patients; (2) by December 2013, ∼ 10–20%; and (3) by December 2014, ∼5% (Naidu KK (HHTCP Lead, 2016)).

As per 2015 guidelines, pregnant women are asked to present for follow-up antenatally six weekly until delivery or more frequently for complicated pregnancies.^9^ Women presenting in labour should be counselled and HIV tested during the first stage of labour and offered routine PMTCT interventions. If not possible, counselling and testing should be offered postpartum. The mother should be counselled on feeding practices and the infant should be tested. Further, women should be started on lifelong ART before discharge and have creatinine and CD4 count checked at the 3 to 6-day postpartum visit. Follow-up visits are aligned with the infant immunisation schedule at 6, 10 and 14 weeks.

### What has been measured?

Data collection for the Hlabisa pregnancy cohort is paper-based with DoH staff collecting routine demographic, clinical and pregnancy data on women attending antenatal services (table 2). The database was designed as an early identification tool for pregnant women requiring further care and to inform clinics of the appropriate actions at each step in the PMTCT cascade. Data flow was not unidirectional as Africa Centre provided action lists, data issues and tracking reports to DoH staff weekly. Additionally, routine PMTCT statistics were reported to clinics at least quarterly when the HHTCP was operational.

**Table 2:** Data collected for all pregnant women in the Hlabisa subdistrict (2010–2014)

Data fields	Variable list
Demographics	Name, national identity number, contact details, date of birth
Clinical visit data	Visit date, antenatal clinic name, other antenatal clinic in close proximity, TB screening, parity, gestational age at first antenatal visit
HIV and related measures	Maternal HIV status at visit; if HIV-infected, prior PMTCT exposure, ART initiation and monitoring bloods including CD4 cell count and HIV viral load, full blood count, liver function tests, renal function tests
Medication history	If HIV-infected, date of start of ART, type of treatment, adherence
Delivery data	Mode of delivery, infant prophylaxis after delivery; if HIV-infected, maternal antiretroviral treatment or prophylaxis taken at delivery
Infant data	Birth weight, birth head circumference, birth length, feeding choice at birth, DNA PCR result at 6 weeks of age

ART, antiretroviral therapy; PMTCT, prevention of mother-to-child transmission; TB, tuberculosis.

Patient monitoring in the HHTCP, including PMTCT care, was as follows: (1) Africa Centre staff responsible for PMTCT data telephoned nurses at the antenatal and HIV clinics to flag abnormal maternal and infant results (HIV tests, CD4 and viral loads), to determine if the appropriate treatment had been provided and if patients were in care; (2) Patients with abnormal blood results were telephoned and asked to return to the clinic for care (Africa Centre staff did not provide blood results telephonically or disclose confidential information); (3) Patients eligible for, or on lifelong ART, who did not return for care were referred to the HHTCP tracking team; and (4) Clinicians in the HHTCP followed up patients on lifelong ART with virological failure, including pregnant women (latest viral load results above 1000 copies/mL after at least 12 months on a standard first-line regimen), offering genotypic resistance testing as part of the HIV Treatment Failure Clinic model and the Southern African Treatment and Resistance Network (SATuRN),^12–14^ changing treatment according to DoH guidelines.^7–11^

Data were electronically captured at the Africa Centre. Data were collected at the following times during routine visits: first antenatal visit, follow-up 1 week after the first visit, week 32 (repeat HIV testing) and infant delivery. The following routine data on HIV care were collected: (1) CD4; (2) HIV staging; (3) clinical tuberculosis screening; and (4) ART prophylaxis or treatment initiation.

Postpartum, data were collected at the 6-week infant visit. Treatment data for HIV-infected pregnant women were linked to the HHTCP cohort, described previously,^4^ to determine details of ART initiation timing, medication history, including ART adherence, baseline and follow-up CD4 and viral loads. All test results were collated into a laboratory database and then imported into the HHTCP database, allowing monitoring of the clinical disease progression of all patients who were initiated on ART.^4^

All data, including monitoring laboratory tests for HIV-infected patients, were as per DoH antenatal and HIV guidelines. We report on the CD4 count according to the 2010 and 2013 HIV guideline eligibility for lifelong ART initiation at 350 cells/mm^3^. 7 11

### Data linkage

Pregnancy data were linked to HHTCP data using names, surnames and identity numbers, or dates of birth when missing identity numbers. Additional information such as cellphone numbers and laboratory test dates were used to verify patient linkage. Trained data capturers linked pregnancy and HIV data sets at capture into the pregnancy database, and through provision of monthly lists generated by a data manager. Password protected data were known only to study investigators and the data team.

#### ACDIS linkage

Individuals in ACDIS are assigned an alphanumeric ‘External Identification Number (DSID)’, which is linked to individual identifiers, including names and/or identity numbers. The ‘External ID’ is linked to an ‘Internal ID’ through a highly protected table, accessible only to the senior data manager. At data capture, an individual's ‘Internal ID’ replaces his ‘External ID’, anonymising all data. The ‘Internal ID’ is added to the HHTCP and pregnancy databases for all ACDIS patients. This ‘Internal ID’ provides the link between the ACDIS, HHTCP and pregnancy databases, allowing de-identified data to be extracted from across databases. An ‘Internal ID’ cannot be used to identify individuals on ACDIS field questionnaires or lists.

## Findings TO DATE

The Hlabisa pregnancy cohort consists of women attending ANC for the first time since January 2010. At the time of data censoring (31 December 2014), the database included 26 520 pregnancies, of whom 25 608 women (96.5%) had HIV test results; 10 469 (40.8%) were HIV-infected and 15 139 were HIV-uninfected (figure 2).

There were 912 (3.4%) women with unknown HIV status excluded from this analysis (indeterminate, missing or refused HIV test). Compared to included women, those excluded were more likely to attend PHC clinics late in pregnancy at or after 30 weeks (p<0.0001). Further, included women were slightly younger at first visit (24 years; IQR=20–29 years) relative to excluded women (26 years; IQR=22–31 years) (data not shown).

HIV-uninfected women were younger (median age 22 years; IQR=19–26 years) than those HIV-infected (median age 27 years; IQR=23–31 years) and presented marginally later to PHC clinics for their first visit (median 28 weeks; IQR=20–38 weeks) versus 26 weeks in HIV-infected women (IQR=18–38 weeks). HIV-uninfected and HIV-infected women had 7833 (51.7%) and 7287 (69.6%) live-born infants delivered at facilities in Hlabisa to date, respectively.

### Clinical characteristics of HIV-infected pregnant women

Of the 10 469 HIV-infected pregnant women, the data of 7634 (72.9%) were linked with the HHTCP database,^4^ to provide detailed information on pregnant women accessing HIV care. Of the 7634 included women, to date 972 (12.7%) had subsequent pregnancies recorded. The linkage with the remaining 27.1% of women was not possible as incomplete national identifying number data made probabilistic linkage between two databases unreliable.

Overall, 25.1% of women (n=1917) were already on lifelong ART at their first ANC visit; 1349 women (17.7%) started lifelong ART within 6 months of the first visit of the current pregnancy.

Before 2013, most women who started on lifelong ART at their first antenatal visit received TDF-based regimens; women on lifelong ART before pregnancy were initiated either on a TDF-based or d4T-based regimen (table 3). There were a further 2347 women (76.4%) who received AZT prophylaxis for PMTCT (table 4). There were 9.6% of HIV-infected women (n=296) who did not receive any ART during pregnancy; of these, 82 women (27.7%) had CD4 ≤ 350 cells/mm^3^ and were eligible for treatment. There may have been several reasons why these women were not started on ART: movement between clinics and not accessing results; transfer out of the subdistrict; or death (0.4%) due to disease progression.

**Table 3:** Characteristics of 7634 HIV-infected pregnant women in the Hlabisa subdistrict from 1 January 2010 to 31 December 2014 by ART status

	Up to 2012			2013 and later		
	On lifelong ART before the first antenatal visit (N=1295)	Started lifelong ART within 6 months of the first antenatal visit (N=912)	Not on lifelong ART (N=3070)	On lifelong ART before the first antenatal visit (N=622)	Started lifelong ART within 6 months first antenatal visit (N=437)	Not on lifelong ART (N=1298)
Median age, years	30 (26–34)	27 (23–31)	25 (21–29)	31 (27–35)	25 (22–30)	26 (22–31)
GA at first visit, weeks						
<12	68 (5.3)	55 (6.0)	177 (5.8)	54 (8.7)	43 (9.8)	59 (4.6)
12–24	363 (28.0)	473 (51.9)	1108 (36.1)	214 (34.4)	240 (54.9)	415 (32.0)
25–37	209 (16.1)	165 (18.1)	631 (20.6)	98 (15.8)	78 (17.9)	200 (15.4)
>37	319 (24.6)	57 (6.3)	436 (14.2)	174 (28.0)	10 (2.3)	416 (32.1)
Missing	336 (26.0)	162 (17.8)	718 (23.4)	82 (13.2)	66 (15.1)	208 (16.0)
Median baseline CD4 count (IQR), cells/mm	166 (105–233)	235 (162–300)	431 (321–568)	187 (125–274)	440 (299–629)	492 (357–656)
Missing	60	37	1290	53	80	340
Baseline CD4 count						
≤350 cells	1179 (91.0)	827 (90.7)	532 (17.3)	513 (82.5)	132 (30.2)	184 (14.2)
>350 cells	56 (4.3)	48 (5.3)	1244 (40.5)	56 (9.0)	225 (51.5)	774 (59.6)
Missing	60 (4.6)	37 (4.1)	1290 (42.0)	53 (8.5)	80 (18.3)	340 (26.2)
On TB treatment at initiation						
Latest drug regimen						
TDF-based	872 (67.3)	768 (84.2)	–	480 (77.2)	437 (100)	–
Stavudine-based	335 (25.9)	16 (1.8)	–	85 (13.7)	–	–
AZT-based	50 (3.9)	79 (8.7)	–	23 (3.7)	–	–
Unknown	38 (2.9)	49 (5.4)	–	34 (5.5)	–	–
Status						
Active	1076 (83.1)	724 (79.4)	1670 (58.5)	560 (90.0)	408 (93.4)	958 (73.8)
Deceased	12 (0.9)	12 (1.3)	10 (0.4)	1 (0.2)	–	4 (0.3)
Loss to follow-up	169 (13.1)	155 (17.0)	1172 (41.0)	54 (8.7)	29 (6.6)	240 (18.5)
Transfer out	38 (2.9)	21 (2.3)	4 (0.1)	7 (1.1)	–	–
Missing			214			96 (7.4)

ART, antiretrovival therapy; AZT, zidovudine; GA, gestational age; TDF, tenofovir; TB, tuberculosis.

**Table 4:** PMTCT regimens of pregnant women in the Hlabisa subdistrict who were not on lifelong ART from 1 January 2010 to 31 December 2014

ART prophylaxis	Up to 2012 (N=3070)	2013 and later (N=1298)
*AZT	2347 (76.4)	186 (14.3)
TDF+FTC+EFV	–	967 (74.5)
None	296 (9.6)	95 (7.3)
Missing	427 (13.9)	50 (3.9)

*Inclusive of either sd-NVP, sd-FTC or both.

ART, antiretroviral therapy; AZT, zidovudine; EFV, efavirenz; FTC, emtricitabine; NVP, nevirapine; PMTCT, prevention of mother-to-child transmission; TDF, tenofovir.

Post-2013, most women established on ART before their first antenatal visit were on TDF-based regimens. Further, the proportion of women who were initiated on d4T regimens (13.7%) before their first visit were less than in prior years (25.9%), reflecting the phased implementation plan for ART, with pregnant women prioritised to start triple ART.^15^ Moreover, relative to earlier years, there was an increase in the proportion of women on established ART before their first visit, and initiated on ART within 6 months of the first visit. All women who were newly initiated on ART within 6 months of their first antenatal visit were on TDF-based regimens. Of the 1298 women who were not on lifelong ART, most were started on the fixed dose combination of TDF+FTC+EFV; and 14% received AZT prophylaxis (table 4). The proportion of pregnant women who did not receive ART prophylaxis or were missing data on treatment decreased post-2013 versus earlier years.

The median maternal age of women not on ART or initiating ART within 6 months of the first visit was younger than that of women already on lifelong ART at pregnancy diagnosis (table 3). Overall, the median gestational age at first visit was 23 weeks (IQR=17–37 weeks). Across the years and irrespective of ART initiation, a low proportion of women had a first antenatal visit before 12 weeks. Despite HIV guideline revisions emphasising the importance of early attendance, over 15% of women attended their first antenatal visit in the third trimester at 25 to 36 weeks gestation. Unexpectedly, there were over 20% of women who were already on lifelong ART with a first antenatal visit after 37 weeks gestation, both pre-2013 and post-2013; this may suggest women on lifelong ART had their pregnancy managed while attending the clinic for HIV treatment. However, this was not standard and women on established lifelong ART were usually referred to the ANC for pregnancy care. While the proportion of women who were newly initiated on ART within 6 months of pregnancy after 37 weeks fell post-2013, more women not on lifelong ART attended their first antenatal visit after 37 weeks relative to earlier years. This may be aligned with guideline revisions to start pregnant HIV-infected women on ART as soon as possible, or reflect an improvement in data collection with less missing data on gestational age post-2013.

In general, women who started lifelong ART within 6 months of pregnancy were in better health at ART initiation with a higher median baseline CD4 count, particularly after guidelines revisions in 2013 (table 3). Viral load monitoring was inadequate during pregnancy, with only 24.2% (n=739) of the women on lifelong ART overall having a viral load test recorded 6 months before or after the first antenatal visit. Of those with viral loads, 12.0% (n=89) had virological failure (ie, viral load ≥1000 copies/mL) within 6 months of their first antenatal visit.

Overall, preliminary analysis suggests that follow-up in the HIV programme was over 80% in women on lifelong ART (n=2768; 80%), at 6 weeks postpartum. Though the proportion of women not on lifelong ART who did not return for care declined in later years versus earlier years, and is likely to be an indicator of better adherence to triple ART prophylaxis post-2013, there were still over 15% of women not in care post-2013.

## Discussion

Data from the Hlabisa pregnancy cohort indicate the low proportion of women who attended their first ANC visit early in the first trimester. Early attendance is particularly important given the current PMTCT guidelines to provide triple ART to HIV-infected women from the first antenatal visit for maternal and infant health. The younger age of HIV-uninfected pregnant women compared with those HIV-infected in this cohort is similar to that of a Sowetan study.^16^ A possible explanation may be that pregnancy rates decline with HIV disease progression.^17^ However, given the expanded ART use in pregnant women which has been associated with higher pregnancy rates,^18^ another likely scenario is that HIV-infected women may be accessing contraception and delaying pregnancy as they progress through the HIV treatment cascade as reported in another Africa Centre study using surveillance data.^19^

The improvement in PMTCT coverage and patient retention post-2013 supports the feasibility and acceptability of the 2013 PMTCT revisions. While Malawi reported an ART coverage increase of ∼49%,^20^ the ART coverage progression in our study was more conservative. These findings should be contextualised by successes within the HHTCP with high ART coverage; by July 2011, ∼37% of all HIV-infected patients were on lifelong ART;^6^ by 2012, over 50 000 patients were enrolled with 25 000 individuals initiated on lifelong ART.^6^ ^12^ Our study indicates that over 65% of women were established on ART before pregnancy and newly initiated on ART within 6 months of the pregnancy pre-2013. These findings reinforce South Africa's commitment to reduce vertical HIV transmission and improve maternal health and child survival consistent with international priorities.^21^ ^22^

Prior studies before Option B implementation have reported ∼25–50% of patients of women on ART at delivery lost to care within 6 months postpartum.^23–25^ While the proportion of women on lifelong ART retained in care in this study was high, the proportion of women initiated on interrupted triple ART prophylaxis post-2013 was higher than the attrition rate in the DREAM cohort of pregnant women in Cameroon initiated on Option B (92.6% at 6 months);^26^ our study reports follow-up status at 6 weeks postpartum and it is possible that retention may deteriorate over time. In Malawi, ∼17% of pregnant women initiated on Option B+ were lost to care by 6 months; most women were lost to follow-up on the day of HIV testing and ART initiation.^27^ These results emphasise the importance of treatment literacy, particularly as pregnant women start ART on higher CD4 counts.

Our finding that 25% of women had viral load testing highlights a common problem in resource poor settings,^28^ and encourages policymakers to seek strategies to overcome this challenge. While pregnancy viral load monitoring may have improved since the latest HIV guideline release,^9^ monitoring of virological failure is necessary to minimise PMTCT leakages that may prevent elimination of paediatric HIV. In a population-based survey including South African data, viral loads above 1000 copies/mL remained undiagnosed in pregnant and breast feeding women.^29^ Virological failure in pregnant women on ART underscores the importance of understanding the different pathways of pregnancy effect, including physiological, hormonal, drug pharmacokinetics and behavioural factors,^30^ as the risk of drug resistance increases as more women on ART become pregnant. Moreover, it is important to engage mothers as partners in their health since it is clear that antiretroviral access does not translate to adherence where mothers are not empowered and engaged.^31–34^ Caution needs to be exercised that budgets that spend enormous amounts on drugs do not get swamped, resulting in diminished resources for the equally important psychosocial elements of management of HIV disease.

Since South Africa has already expanded access to ART and implemented Option B+, it is likely that more women will conceive while on established ART. Strong routine information systems are required to ensure linkage to care, treatment and disease progression monitoring. In future studies, we will link data presented in this study to the Africa Centre surveillance system, providing us with an appropriate denominator for the pregnant population and minimising the challenges posed by double counting in routine systems, necessary for using routine data from PMTCT programmes for ‘HIV surveillance’ as recommended by the WHO.^35^ Additionally, since the pregnancy database was used for the active PMTCT care monitoring, we were able to inform DoH staff of the quality and effectiveness of their services and capacitate staff to improve their routine data. At a policy level, evidence-based decisions, including data from this study, can improve resource allocation and healthcare performance, particularly as South Africa expands access to Option B+. The centralised HIV and pregnancy database in this rural health setting, including routine data, has allowed us to assess maternal HIV status and ART guidelines over time, and is complementary to routine health information systems in South Africa, including the District Health Information System and the Three Interlinked Electronic Register (Tier.Net) to monitor ART provision. As Tier.Net evolves into an active monitoring system for HIV care, the lessons we have learnt can advance understanding of data issues, patient challenges and possible solutions. Moreover, since the HHTCP includes directly imported laboratory data,^4^ the potential for information bias is lessened as we verify routine data of pregnant women in HIV care.

This cohort is generally representative of the Hlabisa subdistrict with data on pregnant women attending all clinics in the area up to 2012, and thereafter still included two of the busiest antenatal clinics in the subdistrict. Moreover, the HIV prevalence reported in this study is comparable to the 2012 antenatal HIV prevalence in the uMkhanyakhude district (35.2%; 95% CI 29.4−41.5).^36^ We are currently conducting analyses in terms of birth outcomes. We also plan a series of analyses to: (1) determine HIV acquisition risk during pregnancy and postpartum; (2) determine the impact of HIV and ART on birth outcomes; (3) examine the effect of pregnancy on virological response to ART; and (4) characterise the effect of sequential pregnancies on access to clinical care and response to prolonged ART and birth outcomes.

### Strengths and limitations

The main strengths of this cohort include its size and the ability to model the impact of the HIV programme on the population due to detailed, longitudinal information available about the community and the linkage between clinical and population data. Accurate characterisation of the cohort will provide an understanding of the determinants of pregnancy outcomes, and implications for service delivery in a typical HIV hyperendemic rural setting, which is likely to be generalisable to other resource-limited settings in South Africa.

One of the main challenges common to cohorts of HIV-infected pregnant women is the attrition rate.^24^ ^37^ ^38^ High migration rates in the Hlabisa subdistrict may limit postpartum assessment of long-term maternal outcomes in this cohort.^39^ During the HHTCP, Africa Centre and DoH staff collaborated to follow-up patients lost to care. It is crucial to follow-up HIV-infected mothers in order to ensure effective ART delivery for maternal and infant health; assess maternal adherence and disease progression; and support safe infant feeding. Using linked population data sets, the pregnancy outcomes of women not retained in care may be determined, and through sensitivity analysis, factors related to poor attrition will be characterised. The clinical pregnancy data will also be used to validate the general health surveys on pregnancy and contraception in the surveillance area, providing sensitivity and specificity estimates of reporting of pregnancy within the Africa Centre surveillance area. Since routine data were collected, it is possible that pregnancy-related details may be incomplete, resulting in information bias. Data discrepancies were queried with clinic and hospital staff prior to data entry. Pregnancy data collected by nurses and counsellors were also verified against antenatal and delivery registers at clinics and the hospital.

### Collaboration

Requests for access to this pregnancy cohort should be directed to the Africa Centre's Helpdesk (help@africacentre.ac.za) with a ‘Research Dataset Request’ in the subject line of the email. A data access agreement will be requested from the researcher and is submitted to the applicable data custodian. The data user will be notified once access approval is granted.

## References

[1] Paintsil E, Andiman WA Update on successes and challenges regarding mother-to-child transmission of HIV. Curr Opin Pediatr 2009;21:94–101. 10.1097/MOP.0b013e32831ec353 19242245 PMC2650837

[2] Barker PM, Mphatswe W, Rollins NC Antiretroviral drugs in the cupboard are not enough: the impact of the health systems’ performance on mother-to-child transmission of HIV. J Acquir Immune Defic Syndr 2011;56:e45–8. 10.1097/QAI.0b013e3181fdbf20 21084998

[3] National Department of Health, South Africa. Clinical guidelines: PMTCT (Prevention of Mother-to-Child Transmission) 2010 http://www.hiv911.org.za/wp-content/uploads/2010/04/2010-PMTCT-Guidelines.pdf (accessed June 2016).

[4] Houlihan CF, Bland RM, Mutevedzi PC Cohort profile: Hlabisa HIV Treatment and Care Programme. Int J Epidemiol 2011;40:318–26. 10.1093/ije/dyp402 20154009 PMC3195268

[5] Tanser F, Hosegood V, Bärnighausen T Cohort profile: Africa Centre Demographic Information System (ACDIS) and population-based HIV survey. Int J Epidemiol 2008;37:956–62. 10.1093/ije/dym211 17998242 PMC2557060

[6] Tanser F, Bärnighausen T, Grapsa E High coverage of ART associated with decline in risk of HIV acquisition in rural KwaZulu-Natal, South Africa. Science 2013;339:966–71. 10.1126/science.1228160 23430656 PMC4255272

[7] Republic of South Africa, Department: Health. The South African Antiretroviral Treatment Guidelines 2013:1–21. http://www.sahivsoc.org/upload/documents/2013. ART Treatment Guidelines Final 25 March 2013.pdf (accessed June 2016).

[8] World Health Organization HIV/AIDS Programme. Use of antiretroviral drugs for treating pregnant women and preventing HIV infections in infants. Executive Summary 2012 http://www.who.int/hiv/PMTCT_update.pdf (accessed June 2016).

[9] National Department of Health SA. National Consolidated Guidelines For The Prevention Of Mother-to-Child Transmission of HIV (PMTCT) And The Management of HIV In Children, Adolescents And Adults 2015 http://www.up.ac.za/media/shared/62/ZP_Files/art-guidelines-15052015.zp57683.pdf (accessed June 2016).

[10] National Department of Health SA. Policy and Guidelines for the Implementation of the PMTCT Programme 2008 http://www.doh.gov.za/docs/policy/pmtct-f.html (accessed June 2016).

[11] South African National Ministry of Health. The South African Antiretroviral Treatment Guidelines 2010 http://www.sahivsoc.org/practise-guidelines/national-dept-of-health-guidelines (accessed June 2016).

[12] Lessells RJ, Stott KE, Manasa J Implementing antiretroviral resistance testing in a primary healthcare HIV treatment programme in rural KwaZulu-Natal, South Africa: early experiences, achievements and challenges. BMC Health Serv Res 2014;14:116 10.1186/1472-6963-14-116 24606875 PMC3973961

[13] Manasa J, Lessells R, Rossouw T Southern African Treatment Resistance Network (SATuRN) RegaDB HIV drug resistance and clinical management database: supporting patient management, surveillance and research in southern Africa. Database (Oxford) 2014;2014:bat082 10.1093/database/bat082 24504151 PMC5630899

[14] Lessells RJ, Avalos A, de Oliveira T Implementing HIV-1 genotypic resistance testing in antiretroviral therapy programs in Africa: needs, opportunities, and challenges. AIDS Rev 2013;15:221–9. 24322382 PMC3951902

[15] South African National Department of Health. Updates on Revised Antiretroviral Treatment Guidelines 2013 2013 http://www.sahivsoc.org/practise-guidelines/national-dept-of-health-guidelines (accessed June 2016).

[16] Kaida A, Laher F, Strathdee SA Childbearing intentions of HIV-positive women of reproductive age in Soweto, South Africa: the influence of expanding access to HAART in an HIV hyperendemic setting. Am J Public Health 2011;101:350–8. 10.2105/AJPH.2009.177469 20403884 PMC3020203

[17] Sedgh G, Larsen U, Spiegelman D HIV-1 disease progression and fertility in Dar es Salaam, Tanzania. J Acquir Immune Defic Syndr 2005;39:439–45. 16010167 10.1097/01.qai.0000148529.58963.83

[18] Myer L, Carter RJ, Katyal M Impact of antiretroviral therapy on incidence of pregnancy among HIV-infected women in Sub-Saharan Africa: a Cohort Study. PloS Med 2010;7:e1000229 10.1371/journal.pmed.1000229 20161723 PMC2817715

[19] Raifman J, Chetty T, Tanser F Preventing unintended pregnancy and HIV transmission: effects of the HIV treatment cascade on contraceptive use and choice in rural KwaZulu-Natal. J Acquir Immune Defic Syndr 2014;67(Suppl 4):S218–27. 10.1097/QAI.0000000000000373 25436821 PMC4251916

[20] Barr B, Mhango E, Tenthani L Uptake and retention in Malawi Option B+ PMTCT program: lifelong ART for all HIV+ pregnant or lactating women. *14th Conference on retroviruses and Opportunistic Infections*. Atlanta: 2013.

[21] UNAIDS. 2015 Progress Report on the Global Plan towards the elimination of new HIV infections among children and keeping their mothers alive. 2015 http://www.unaids.org/en/resources/documents/2015/JC2774_2015ProgressReport_GlobalPlan (accessed Oct 2016).

[22] United Nations. The Millennium Development Goals Report 2008. 2008 http://www.un.org/millenniumgoals/2008highlevel/…/mdg reports/MDG_Report_2008_… (accessed June 2016).

[23] Phillips T, Thebus E, Bekker LG Disengagement of HIV-positive pregnant and postpartum women from antiretroviral therapy services: a cohort study. J Int AIDS Soc 2014;17:19242. 25301494 10.7448/IAS.17.1.19242 PMC4192834

[24] Kurewa EN, Kandawasvika GQ, Mhlanga F Realities and challenges of a five year follow-up of mother and child pairs on a PMTCT program in Zimbabwe. Open AIDS J 2011;5:51–8. 10.2174/1874613601105010051 21760874 PMC3134989

[25] Geddes R, Giddy J, Butler LM Dual and triple therapy to prevent mother-to-child transmission of HIV in a resource-limited setting—lessons from a South African programme. South African Med J 2011;101:651–4. 21920158

[26] Altan AMD, Taafo F, Fopa F An assessment of option B implementation for the prevention of mother to child transmission in Dschang, Cameroon: results from the DREAM (Drug Resource Enhancement against AIDS and Malnutrition) cohort. Pan Afr Med J 2016;23:72 10.11604/pamj.2016.23.72.7958 27217896 PMC4862786

[27] Tenthani L, Haas A, Tweya H Roll-out of universal antiretroviral therapy for HIV infected pregnant and breastfeeding women (‘Option B+’) in Malawi: factors influencing retention in care. *5th International Workshop on HIV Paediatrics*. Kuala Lumpur, Malaysia: 2013.

[28] Sigaloff KC, Hamers RL, Wallis CL Unnecessary antiretroviral treatment switches and accumulation of HIV resistance mutations: two arguments for viral load monitoring in Africa. J Acquir Immune Defic Syndr 2011;58:23–31. 10.1097/QAI.0b013e318227fc34 21694603

[29] Maman D, Huerga H, Etard JF Most breastfeeding women with high viral load are still undiagnosed in sub-Saharan Africa. *Conf. Retroviruses Opportunistic Infect*. 2015.

[30] Westreich D, Cole SR, Nagar S Pregnancy and virologic response to antiretroviral therapy in South Africa. PLoS ONE 2011;6:e22778 10.1371/journal.pone.0022778 21829650 PMC3149058

[31] Nachega JB, Uthman OA, Anderson J Adherence to antiretroviral therapy during and after pregnancy in low-income, middle-income, and high-income countries: a systematic review and meta-analysis. AIDS 2012;26:2039–52. 10.1097/QAD.0b013e328359590f 22951634 PMC5061936

[32] Bailey H, Thorne C, Malyuta R Adherence to antiretroviral therapy during pregnancy and the first year postpartum among HIV-positive women in Ukraine. BMC Public Health 2014;14:993 10.1186/1471-2458-14-993 25248469 PMC4180980

[33] Nicastri E, Ivanovic J, Signore F Antiretroviral therapeutic drug monitoring in HIV-infected pregnant women: maternal immunovirological outcome at delivery and during the 18 month follow-up period. Curr HIV Res 2012;10:606–13. 22762419 10.2174/157016212803306014

[34] Mepham S, Zondi Z, Mbuyazi A Challenges in PMTCT antiretroviral adherence in northern KwaZulu-Natal, South Africa. AIDS Care 2011;23:741–7. 10.1080/09540121.2010.516341 21293987

[35] UNAIDS/WHO Working Group on Global HIV/AIDS and STI Surveillance. Guidelines for assessing the utility of data from prevention of mother-to-child transmission (PMTCT) programmes for HIV sentinel surveillance among pregnant women 2013: http://apps.who.int/iris/bitstream/10665/85512/1/9789241505611_eng.pdf (accessed June 2016).

[36] South African National Department of Health. The 2012 National Antenatal Sentinel HIV and Herpes Simplex type-2 prevalence Survey, South Africa 2013 http://www.health-e.org.za/wp-content/uploads/2014/05/ASHIVHerp_Report2014_22May2014.pdf (accessed June 2016).

[37] Geldsetzer P, Yapa HMN, Vaikath M A systematic review of interventions to improve postpartum retention of women in PMTCT and ART care. J Int AIDS Soc 2016;19:20679. 27118443 10.7448/IAS.19.1.20679 PMC4846797

[38] Dzangare J, Takarinda KC, Harries AD HIV testing uptake and retention in care of HIV-infected pregnant and breastfeeding women initiated on ‘Option B+’ in rural Zimbabwe. Trop Med Int Health 2016;21:202–9. 10.1111/tmi.12637 26555353

[39] McGrath N, Eaton JW, Newell ML Migration, sexual behaviour, and HIV risk: a general population cohort in rural South Africa. Lancet HIV 2015;2:e252–9. 10.1016/S2352-3018(15)00045-4 26280016 PMC4533230

